# Implementing mainstream germline genetic testing in breast cancer across Europe

**DOI:** 10.1038/s44276-025-00202-w

**Published:** 2026-02-03

**Authors:** Eduard Pérez-Ballestero, Sagal Ahmed Shire, Mateja Krajc, Arvīds Irmejs, Lenka Foretová, Sophie Frank, Tiina Kahre, Thomas van Overeem Hansen, Linetta Koppert, Anna Lena Burgemeister, Marc Tischkowitz, Judith Balmaña, Svetlana Bajalica-Lagercrantz

**Affiliations:** 1 Medical Oncology Department, Hospital Vall d’Hebron, Vall d’Hebron Campus, Universitat Autònoma de Barcelona, Barcelona, Spain; 2 Department of Clinical Genetics, Sout West Thames Centre for Genomics, St. George’s University Hospital, London, UK; 3 Department of Clinical Cancer Genetics, Institute of Oncology Ljubljana, Ljubljana, Slovenia; 4 Pauls Stradins Clinical University Hospital, Riga, Latvia; 5 Masaryk Memorial Cancer Institute, Brno, Czech Republic; 6 Institut Curie, Paris, France; 7 Genetics and Personalized Medicine Clinic, Tartu University Hospital, and Institute of Clinical Medicine, University of Tartu, Tartu, Estonia; 8 Department of Clinical Genetics, Rigshospitalet, Copenhagen University Hospital, and Department of Clinical Medicine, University of Copenhagen, Copenhagen, Denmark; 9 Erasmus Medical Center, Rotterdam, the Netherlands; 10 MGZ—Medical Genetics Center, Munich, Germany; 11 Department of Medical Genetics, National Institute for Health Research Cambridge Biomedical Research Centre, University of Cambridge, Cambridge, UK; 12 Department of Oncology-Pathology, Karolinska Institutet, and Hereditary Cancer Unit, Department of Clinical Genetics and Genomics and Theme Cancer, Karolinska University Hospital, Stockholm, Sweden

## Abstract

The implementation of mainstream germline genetic testing in breast cancer patients has both benefits and challenges. Multiple aspects need to be considered for the outline of gene panels and the amount of pre-test genetic counselling. Mainstream genetic testing is mainly performed to stratify patients for targeted treatment. In addition, identification of germline pathogenic variants in cancer risk genes may have surgical implications, consequences for surveillance of other organs at risk of cancer, as well as family implications among relatives at risk. To ensure that patients are well informed, the introduction of mainstream genetic testing performed by non-genetic health care specialists requires an adapted pre-test counselling visit. Here, we review the literature and propose a web-based educational session and pocket guide to support implementation of mainstream testing in oncology practice.

## Introduction

Genetic characterization in cancer patients in the era of personalized treatment is challenging established clinical routines for germline testing. The traditional model of pre- and post-genetic counselling, typically delivered by clinical geneticists or genetic counsellors, is time-consuming and, due to a shortage of trained professionals, it often fails to meet the turnaround times required for decision-making in cancer treatment settings [1, 2]. If the responsibility for genetic counselling and testing remains limited to these professionals, it will not be feasible to address the increasing demand for germline genetic testing oncology in the coming years [3].

To address this gap, a mainstream genetic testing model has been proposed and introduced in several European cancer clinics. In this model, non-genetic healthcare professionals (NGHCPs)—such as oncologists, surgeons, and clinical specialist nurses—take on the roles of pre-test counselling and test ordering. This approach has demonstrated high uptake and acceptability among patients and providers [4]. While Hamilton’s model improves access and reduces turnaround time, it relies heavily on oncologists' ability to integrate genetic considerations into routine care. In contrast, our proposal focuses not only on feasibility but also on the co-development of adaptable workflows with clinicians, supported by web-based tools and practical recommendations. Rather than evaluating an existing model, we offer a structured framework based on expert knowledge and a targeted review of current strategies to support integration across diverse clinical settings. Mainstreaming refers to a healthcare process where the main intent of genetic testing is to stratify treatment, diagnosing hereditary cancer risk while simultaneously informing therapeutic decisions—i.e., theragnostic application [5, 6].

However, this shift is often implemented with limited counselling and without comprehensive information about the potential to identify germline pathogenic variants (gPV) in cancer predisposition genes. As a result, patients may undergo genetic testing without being fully informed about its broader implications, including the potential need for additional surveillance of other organs or the importance of cascade testing for relatives who may also be at genetic risk. These issues raise ethical and practical concerns about the adequacy of information provision and patients’ rights to informed decision-making.

In addition to logistical considerations, mainstream genetic testing presents clinical challenges related to the selection of appropriate gene panels, the interpretation of variants, and the determination of clinical actionability of germline pathogenic variants (gPVs) when handled by non-genetic healthcare professionals (NGHCPs). To further explore these issues, we conducted a narrative literature review focused on the implementation of mainstream genetic testing in breast cancer care. Most relevant topics were the decision-making processes, barriers and facilitators to integration in clinical settings, and the educational requirements of NGHCPs. Insights from this review informed the development of two practical tools within the framework of the European Reference Network for Genetic Tumor Risk Syndromes (ERN GENTURIS): a web-based educational session and a concise pocket guide, both designed to support the effective and responsible application of mainstream genetic testing in oncology practice.

## Literature search strategy and review methodology

This manuscript presents a narrative literature review conducted to identify key challenges and facilitators associated with the implementation of mainstream germline genetic testing in breast cancer care, with a particular focus on decision-making processes involving non-genetic healthcare professionals (NGHCPs). The overarching aim was to extract common themes from the literature that could inform the development of targeted educational and clinical support tools to enhance the integration of genetic testing into oncology practice.

**Fig. 1:**
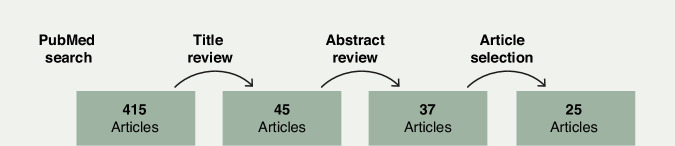
The outline of the search process based on the initial Pub Med search terms (germline[title] OR genetic[title]) AND (Ovarian OR Pancreatic OR Breast OR Colorectal) AND Decision-making identified 415 articles. After a selection process of estimated relevance based on the Title, Abstract and Full paper, a total of 25 papers were included.

A systematic search of the PubMed database was performed on November 28, 2023. The search strategy employed MeSH-based terms applied to article titles using the following Boolean string: (germline [title] OR genetic [title]) AND (ovarian OR pancreatic OR breast OR colorectal) AND decision-making. This search yielded a total of 415 articles published between 1996 and 2023 (Fig. 1).

### Selection and inclusion criteria

Initial screening was carried out independently by four reviewers (SBL, JB, SAS, and EPB), who assessed title relevance. Articles selected by at least two reviewers were advanced to abstract review. Abstracts were assessed based on predefined inclusion criteria: (1) a focus on mainstream genetic testing, (2) relevance to clinical decision-making, (3) applicability to breast cancer care, and (4) involvement of NGHCPs in the genetic testing process. Articles exclusively describing the traditional genetic counselling model—i.e., genetic testing coordinated solely by clinical geneticists or certified genetic counsellors—were excluded.

Of the 415 articles initially identified, 45 met the criteria for abstract review. Abstracts were co-reviewed in pairs (23 by JB and EPB; 22 by SBL and SAS), resulting in 37 articles selected for full-text evaluation. Following full-text review, 25 articles were deemed to meet the eligibility criteria for inclusion in this review. Additional references were incorporated where relevant to provide conceptual context and strengthen the discussion.

### Thematic analysis approach

A qualitative thematic analysis was conducted on the final selection of articles. Each article was reviewed in full by at least two authors using a standardized data extraction template, which captured key information such as study design, population characteristics, geographic region, clinical setting, and major findings. Thematic categories were developed inductively based on recurring content across the included studies.

From the analysis of the selected literature, several recurrent themes emerged that collectively illustrate the multifaceted nature of mainstream genetic testing in oncology.

One relevant issue was the limited availability of time within standard oncology consultations, which often constrains the depth and quality of genetic counselling that can be offered to patients. This challenge is particularly acute when clinicians are expected to integrate complex genetic information into already time-pressured clinical consultations.

On the other hand, there is the difficulty many NGHCPs face in delivering accurate pre-test information and interpreting post-test results. These professionals often lack formal training in genetics, which can interfere with their ability to effectively communicate the implications of testing, including risks to family members and recommendations for surveillance or preventive measures. Another recurring theme was the use of genetic testing primarily as a tool for treatment stratification, highlighting its growing theragnostic utility. While this approach enhances the personalization of care, it also risks overlooking broader hereditary implications if testing is indicated within the aim of a therapeutic context.

The literature review also revealed uncertainties regarding which genes should be included in testing panels and what clinical criteria should guide the decision to test. Variability in panel composition and testing thresholds across institutions was frequently noted, raising concerns about consistency and clinical validity.

Finally, there was a strong emphasis on the educational needs of NGHCPs. Many studies reported gaps in knowledge and confidence among these providers, along with a need for practical, accessible support tools. Structured training programs, brief educational modules, and decision aids were identified as potentially effective strategies to enhance competence and ensure the responsible implementation of mainstream genetic testing in routine oncology care.

The emergent themes informed both the synthesis of current literature and the rationale for developing supportive tools aimed at improving the quality and reach of mainstream genetic testing. These tools, developed by the authors within the framework of the European Reference Network for Genetic Tumor Risk Syndromes (ERN GENTURIS), include a web-based educational module and a pocket guide designed to facilitate appropriate test delivery and patient communication by NGHCPs.

A detailed overview of the included studies and their thematic contributions is available in the Supplementary Table.

## Results of literature search

The following headlines were selected based on the most relevant topics identified in the literature, as well as those aspects considered to be of primary importance by the experts conducting the review, following the implementation of mainstreaming in daily clinical practice.

### Perceived lack of time and training to provide an adequate pre-test genetic counselling

Our literature review identified several key barriers to the implementation of mainstream germline genetic testing [7], which emerged from our analysis of the literature, not pre-determined by the authors. These barriers include the shortage of qualified genetic counselors [8], as well as the lack of knowledge among non-genetic healthcare professionals (NGHCPs) regarding pre-test consent and the disclosure of results in terms of cancer risk assessment, actionability, and interpretation of variants’ pathogenicity [9]. Additionally, there is poor awareness among physicians of best practice guidelines [10].

Pre-test counselling refers to providing patients with information about the purpose of genetic testing, potential outcomes, implications for their health and family members, and obtaining informed consent. Post-test counselling occurs after the results are received, focusing on interpreting the results, discussing clinical implications, providing emotional support, and recommending next steps in medical management and family testing.

Although efforts have been made to train NGHCPs in genetic counselling, a significant barrier remains: a perceived lack of time during appointments to provide comprehensive counselling services [11]. However, a more recent systematic review [12] showed that NGHCPs considered the required time investment acceptable, despite the increased workload. The review concluded that implementing mainstream genetic testing is feasible for NGHCPs, as it requires only a modest amount of additional time during appointments and for training. Compared to standard genetic counselling, which typically takes 40–50 min, NGHCP-delivered counselling is significantly shorter, averaging 8–20 min [13]. Furthermore, the turnaround time for results was reduced by approximately 85% [14]. These findings support the feasibility of mainstreaming as a model for delivering genetic testing, especially when immediate results are crucial for treatment decision-making.

In the context of NGHCPs delivering genetic services, concerns have been raised about the lack of adherence to established clinical guidelines for providing adequate genetic counselling [15]. Instances have been reported where NGHCPs fail to conduct comprehensive risk assessments or ensure informed consent for germline genetic testing. This has resulted in negative outcomes, including incorrect test orders, adverse emotional consequences, improper medical management, misinterpretation of results, and unnecessary surgeries. Moreover, partial or non-compliance with clinical guidelines may expose NGHCPs to legal risks [16]. To address these challenges, evidence suggests that targeted training programs for NGHCPs could enhance their ability to deliver proper genetic services [13].

### Challenges in genetic counselling and cancer risk communication

Effective genetic counselling in oncology relies on two fundamental and complementary competencies: a solid understanding of cancer genetics and proficient communication skills. A solid understanding of cancer genetics allows healthcare providers to identify when genetic testing is appropriate and to explain the broader implications of test results, including how they may affect treatment decisions and the potential risks for family members. Communication competence, meanwhile, is essential for engaging in complex discussions with patients and their families, accurately interpreting and explaining genetic test outcomes, offering personalized cancer risk estimations, and outlining strategies for surveillance and risk reduction [10, 11].

In the pre-test context, the primary goal of genetic counselling is to equip patients with sufficient information to support informed decision-making [17]. Research has demonstrated that patients, particularly women diagnosed with breast cancer, often incorporate genetic information into their treatment decisions when rapid genetic testing is made available at diagnosis [18].

Despite its importance, the process of delivering genetic counselling remains a key challenge in the implementation of mainstream genetic testing. NGHCPs have reported limited confidence in their ability to provide pre- and post-test counselling and have consistently emphasized the need for additional training, particularly in post-test result interpretation and communication [11]. Communicating complex cancer risk information in a clear and sensitive manner was identified as a specific area where many NGHCPs expressed a desire for further education [19, 20]. In response to the shortage of clinical geneticists and genetic counsellors, as well as the growing demand of genetic testing, several alternative models of genetic counselling delivery have emerged [21]. These models aim to maintain the quality of patient care while improving access and efficiency. For instance, in some cancer centers, genetic counsellors have been integrated directly into oncology services to support NGHCPs. In other cases, NGHCPs—when appropriately trained—have assumed responsibility for initial counselling under national legal frameworks [3].

An illustrative example of a hybrid model is seen at the Royal Marsden Hospital (London, UK), where NGHCPs deliver mainstream testing with structured support from clinical geneticists. Results are reviewed centrally: if no pathogenic variant is detected, a standardized results letter and information sheet are sent to the patient. However, when a pathogenic variant or a variant of uncertain significance (VUS) is identified, the patient is referred to the Clinical Genetics Department for further counselling [22]. Similarly, Scheinberg et al. [23] describe a model in which even patients with negative results, but a strong family history, are referred for additional assessment by genetic specialists to ensure accurate risk evaluation.

These evolving service models underscore the importance of structured collaboration between oncology and genetics and highlight the need to ensure that NGHCPs are adequately trained to fulfill their expanding roles in genetic counselling.

### Knowledge on cancer genetics

One concern in mainstream genetic testing is to guarantee the patient’s right to be adequately informed to make an informed decision, including the potential consequences of a genetic test. Since testing in mainstream has repeatedly been reported to be beneficial to cancer management [6, 13], it is important to encourage its implementation, and at the same time improve genetic counselling procedures among NGHCPs to ensure informed patients. NGHCPs have raised some concerns implementing the mainstream testing [11] mainly related to their perceived knowledge in genetics, counselling and their clinic infrastructure. It has been indicated that many NGHCPs have insufficient knowledge in cancer genetics to offer any genetic counselling [9].

Genetic counselling and interpretation of genetic reports by NGHCP was in the past regarded as possibly harmful for patients [10, 24]. The development and implementation of programs to educate the treating physicians about predictive and diagnostic genetics has improved patients’ care [20]. To address the lack of knowledge several educational programs have been implemented, such as mandatory one-to-two-hour in-service presentation led by a hereditary cancer unit [25] or online training programs to raise the main topics related with genetic counselling [5, 25].

Educational programs for oncologists in the context of mainstream genetic testing implementation have been effective, with a significant reduction in perceived barriers, particularly regarding skills, knowledge, and attitudes. Oncologists received training through practical workshops on coordinating genetic testing for ovarian cancer patients. Despite these advancements, 58% of professionals reported not always conducting *BRCA* testing according to guidelines, indicating persistent barriers that are not fully addressed by the current training. This suggests that while the training is valuable, additional interventions are needed to address the remaining challenges.

Competency in mainstream genetic testing is critical as it becomes increasingly integrated into oncology care. For oncology healthcare professionals (NGHCPs) to effectively offer genetic testing, it is essential that they possess not only the technical skills to order and interpret tests, but also the capacity to engage in comprehensive genetic counseling and communicate complex results. Defining and standardizing competency in this area would help ensure consistent, high-quality care across diverse practice settings. Moreover, the recognition of genetic testing as a core competency could lead to the development of quality assurance programs, supporting continuous professional development.

## Discussion

### Genetic testing for treatment stratification in breast cancer patients

In breast cancer, germline genetic testing results may impact on surgical, neo- and adjuvant systemic oncological treatment as well as on radiotherapy [26]. Clinical handling therefore requires faster turnaround time of genetic testing through a mainstream pipeline. The improved lead time is of particular importance, not only in patients with a metastatic disease and where targeted oncological treatment is considered, but also when considering breast cancer surgery as the initial treatment [27], and when radiotherapy is better avoided (i.e., in *TP53*-carriers). Breast surgeons strive for breast preserving surgery [28]. However, if a breast cancer patient is found to be a carrier of a high-risk pathogenic variant in a breast cancer risk gene it may be beneficial to perform concomitant ipsilateral risk reducing mastectomy and reconstruction, instead of an initial breast sparing surgery followed by a second risk reducing surgery, thus minimizing the number of surgical interventions needed. Notably, the increased usage of neoadjuvant chemotherapeutic treatment enables slightly longer turnaround times before surgical interventions. Another approach is the tumor-first screening commonly used in mainstream testing in ovarian cancer, in which therapeutical decisions are often independent of germline status. This allows a separation between treatment predictive testing and hereditary aspects. If tumor screening does not identify a PV in a high breast cancer risk gene, surgery could be adjusted accordingly. Patients could then later be offered genetic counselling prior to germline testing. Likewise, in patients where treatment stratifications are independent of germline status, but the pedigree is indicative for genetic workup, the genetic counselling and testing can be performed through the slower traditional pathway for cancer genetics, commonly via clinical genetics units.

### Genetic testing criteria

In a previous survey performed within ERN GENTURIS, we have showed that the familial and individual criteria for breast cancer patients eligible for genetic testing vary in different European countries [29]. We have also observed a tendency for pedigree criteria to diverge towards lower stringency upon introduction of treatment predictive genetic screening [30, 31], allowing more breast cancer patients to access genetic screening irrespective of their family history. For example, the latest National Guidelines in Sweden from 2024 [32], include all patients with triple negative breast cancer (TNBC) for genetic testing while the previous guideline stated TNBC at or below age 60 [33]. This rational is supported by a retrospective study on more than 5000 breast cancer patients, that showed that more than 60% of all identified *BRCA1/2* carriers did not fulfill the traditional screening criteria, and were thus initially missed [34]. A suggestion for minimal criteria for genetic testing in the mainstream delivery model for breast cancer is presented in Table 1, according to criteria approved for targeted therapies and reasonable clinical situations with a high likelihood to identify a germline pathogenic variant that might lead to a risk reduction approach at the time of diagnosis.

**Table 1:** When the genetic result may impact on surgical, neo- and adjuvant therapy, or radiotherapy

Consideration of uni- or bilateral risk reducing mastectomy and reconstruction
Decision on systemic targeted therapy (standard or clinical trial)
Avoidance of radiotherapy
Individual and pedigree criteria (see Table 2)^a^: Known PV in a family Triple negative breast cancer (TNBC) Breast cancer (BC) < 40 years^a^ Male BC Breast and ovarian cancer in the same individual BC patients with a pronounced family history^a^ (relatives with cancer in breast (young onset), ovary, prostate, pancreas) Specific ancestry origin - consider founder PV

^a^Criteria depends on country-specific guidelines; *PV* pathogenic or likely pathogenic variant.

### Gene selection for clinical decision making

We are presenting possible scenarios of gene selection decisions in routine clinical practice with regards to available resources (funding, testing equipment and available trained professionals). In case of clinical decision making in breast cancer, the reasoning for different scenarios of gene selection is explained in Table 2.

**Table 2:** Possible scenarios of gene selection when testing is needed for clinical decision making in breast cancer

STRATEGY INDICATED	WHICH GENES	REASONING	
		PROS	CONS
OPTIMAL SET	HBOC PANEL^a^	Immediately actionable in the therapeutic setting	More VUSes
		+	+
		Surveillance for carriers	Risk of longer turnaround
		+	+
		No need for further genetic assessment in case of negative result	Increases the complexity of genetic counselling
MINIMAL SET	*BRCA1, BRCA2, PALB2 and TP53*	Immediately actionable in the therapeutic setting	If no PV identified, further genetic testing might be needed in case of high probability of PV
		+	
		Surveillance for carriers	
		+	
		Providing genetic testing to a larger number of potential carriers	
		+	
		Fast turnaround time	
		+	
		Less demanding and faster pre-test counselling towards treatment implications of the results	
In the setting of LIMITED RESOURCES	Founder variants in *BRCA1 and BRCA2* (country specific)	Immediately actionable in the therapeutic setting	If no PV identified, further genetic testing might be needed in case of high probability of PV
		+	+
		Surveillance for carriers	Does not take in consideration patients of other ancestry
		+	+
		Providing genetic testing to a larger number of potential carriers	Not optimal if patient has another ancestry than the country of residency
		+	
		Fast turnaround time	
		+	
		Less demanding and faster pre-test counselling towards treatment implications of the results	

*PV* pathogenic or likely pathogenic variant, *VUS* variant of uncertain significance.

^a^Criteria depends on country specific guidelines.

Three different scenarios are presented; the first one would be an optimal gene panel setting, the second one for the national health care systems where resources are less limited, and the third option for health systems with limited resources (minimal set of genes) or very limited and with founder variants.

Optimal gene set testing based on country specific hereditary breast and ovarian cancer (HBOC) panel offers the immediate identification of possible germline findings for treatment planning and optimal surveillance for new primary cancers. In case of not finding a genetic alteration, there is limited need of further genetic assessment if the family history is not indicative for other genetic tumor risk syndromes. However, there is an increased complexity of genetic counselling and risk of longer turnaround time and a higher VUS rate [35].

In the minimal set, instead of full breast cancer gene panel screening, a focus on selected genes (i.e.,* BRCA1, BRCA2, PALB2* and *TP53*) with an immediate implication on precision treatment planning may be used. Nevertheless, in case of negative genetic screening results, further genetic assessment may still be needed if indicated by family history.

Finally, a testing strategy including only country specific founder variants could be feasible with a potential cost benefit in countries with very limited resources and a high prevalence of founder mutations. This strategy might miss non-founder variants, especially when testing residents with an ancestry other than the founder population. As mentioned in the minimal set scenario, in cases of negative screening results, the patient may still have to go through a complementary genetic counselling and screening, especially if the pedigree fulfills the testing criteria for screening with a HBOC panel. In the end, this strategy may result in a more costly procedure.

### Germline testing and aspects of surgery

When considering breast cancer surgery and reconstruction, several aspects merit consideration, such as avoidance of radiotherapy where harmful consequences have been described. For instance, in *TP53*-carriers radiotherapy increases the risk of secondary soft tissue sarcoma in the irradiated field [36, 37], and therefore, if mastectomy can avoid radiotherapy, it should be preferred rather than lumpectomy and radiotherapy. Concerning other high risk gene carriers, as *BRCA1/2*, radiation has not been associated with any increased cancer risk [38]. When adjuvant radiotherapy is not needed, mastectomy and immediate breast reconstruction may therefore be a better option for these women [39]. Indeed, breast reconstruction after radiotherapy is surgically more complex and often leads to less favorable cosmetic results [40]. Decisions concerning contralateral risk-reducing surgery, and their timing, need to be considered multiple aspects such as remaining cancer risk or relapse risk of primary cancer. Therefore, they should be discussed in a multidisciplinary board [41].

### Adaptation of clinical pathways for mainstream germline testing

Due to the increased demand for genetic testing and the demand for faster turnaround time [42] the mainstreaming might be a new delivery model to cope with the current needs prioritizing the testing as a diagnostic test for cancer patients.

The implementation of the pathway depends on different circumstances such as human resources, in-house or outsourcing genetic testing delivery, educational training to NGHCPs or time-consuming actions. Other health care staff such as breast surgeons, gynecologists, oncologists or breast cancer nurse specialist [43] assessing patients on the pre-test genetic assessment would facilitate the implementation of the pathway.

It is crucial to acknowledge that the structure of each health care service may influence the application of procedure. Prior to starting it is important to find out which is the system between the laboratory and clinics. In some cases, it will be necessary to establish an outsourcing genetic testing delivery system to central labs with a high volume of testing compared to the low activity of genetic testing. In these cases, there is a need for consistent and sustainable sample delivery [44].

As mentioned before, NGHCPs should receive basic training enabling them to provide basic information regarding genetic testing. Patients should receive clear and concise information [45], including the reason for the genetic test request and possible results, as well as understanding the personal implications for their treatment or increased risk of other tumors [46]. Additionally, they should be aware that finding a gPV may mean relatives could be at risk and need be to informed. It is important that patients sign an informed consent form covering all aspects discussed during the consultation [47] but this does not apply for worldwide jurisdictional practices, so documentation of the relevant discussion in the medical record could be a reasonable alternative, depending on local laws. Genetic testing should be equally accessible to all patients [48]. There is a significant gap in the literature, particularly regarding equitable access for individuals in rural or remote areas, as well as the challenges of providing culturally competent care to diverse communities.

Additional resources should be given to the patients in case they need support information to help them on decision making [49]. Leaflets or websites could be helpful to synthesize the information given [50].

## Education session and pocket guide

One challenge that appears in mainstream testing is to provide prerequisite information to patients concerning consequences of a genetic analysis with the potential to identify germline variants in cancer risk genes. Healthcare professionals that are treating cancer patients are facing an increasing arsenal of therapeutic options that require deeper understanding in genetics, often with an experience of high clinical workload. The introduction of additional tasks such as pre-test genetic counselling may seem impossible. Our literature review has identified the need of education and support with clinical protocols to ensure informed patients.

**Fig. 2:**
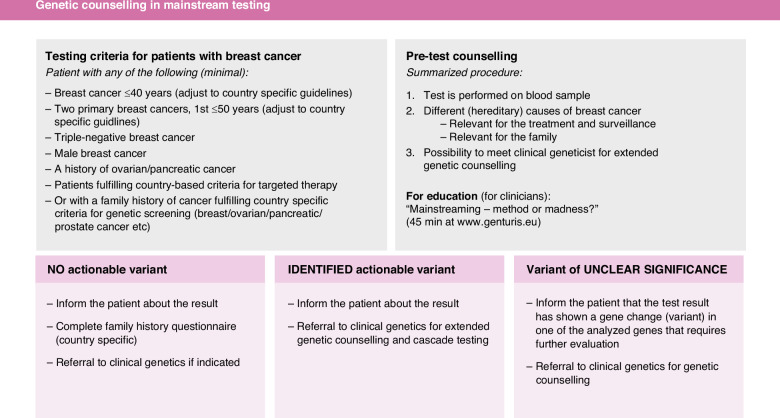
A Pocket guide for health care providers for pre-test genetic counselling in mainstream testing. The testing criteria (first section, upper left) should be adapted to country specific guidelines. A 30 min educational webinar provided by ERN GENTURIS ((ERN GENTURIS Webinar (free of charge): “Mainstreaming – method or madness?” by Marc Tischkowitz and Judith Balmaña; requires a brief registration) is recommended for NGHCP before implementing mainstream genetic testing. *The follow up of the genetic testing result to be adjusted to the available pathways at each hospital (third section, lower)*.

In order to facilitate for the NGHCP to introduce pre-test genetic counselling in their workflow we have designed a pocket guide for genetic pre-test counselling in mainstream testing (Fig. 2). The pocket guide was developed by clinical geneticists and medical oncologists within the European Reference Network on Genetic Tumor Risk Syndromes (ERN GENTURIS, www.genturis.eu.) from thematic group 3 (Hereditary Breast and Ovarian Cancer (HBOC) and task force 4 (Continuous Education, Training & Development), and it was revised by two co-authored genetic counsellors (EPB and SAS). Although it has not been methodically evaluated, the pocket guide was tested in the clinical setting at the Hereditary Cancer Unit at Karolinska University Hospital, Sweden, during 3 months and adjusted according to minor suggestions made by the NGHCP. The Pocket guide is divided into three separate sections, i.e., testing criteria, pre-test counselling procedure as well as result communication and further processing, that can easily be adapted to local circumstances. To meet basic educational needs for NGHCP we have within the ERN GENTURIS network also developed an adjusted educational session that are directed to cancer care providers when introducing genetic testing in mainstream (Mainstreaming – method or madness; https://www.genturis.eu/l=eng/Education-and-training/Webinars/26-4-2023.html) Fig. 2, second section, upper right). Even though the impact of the webinar has not been evaluated, the lecturers and co-authors MT and JB are comparing and contrasting their experiences as a clinical geneticist and oncologist, respectively, of implementing mainstream genetic testing for common hereditary cancer types.

## Conclusion

One challenge in precision medicine is to provide adequate and proportional information to patients concerning the implications of a genetic analysis with the potential to identify germline variants in cancer risk genes. In the mainstream setting, this should be provided by the treating surgeons and oncologists. Our literature review has identified a need of deeper understanding of genetics among NGHCP as well as support material in the daily clinical work to ensure good quality pre-test information to the patients. We therefore present an educational session and a pocket guide for clinical implementation of counselling in mainstream genetic testing. Genetic profiling also challenges the health care system due to increasing costs. Hence, we suggest a rationale for gene panel selection in mainstream testing for countries with different resources and discuss potential consequences with limited gene panels. As mainstream genetic testing is implemented, the quality of cancer risk communication will need to be continuously evaluated to ensure informed patients and guarantee educational level and genetic competence of NGHCP, to reach equity and access of mainstreaming.

## Supplementary information

250925 ERN GENTURIS Thematic Group 3 members - supplementary materials_revisedSept25

Sup Table 1.Final

## Data Availability

No datasets were generated or analysed during the current study.
