# Supplementary material for: Implementing mainstream germline genetic testing in breast cancer across Europe
Source: BJC Rep. 2026 Feb 3;4:3. doi: 10.1038/s44276-025-00202-w (PMC12867965; doi:10.1038/s44276-025-00202-w)
Supplement: Supplementary file 1 — 250925 ERN GENTURIS Thematic Group 3 members - supplementary materials_revisedSept25 [file 44276_2025_202_MOESM1_ESM.docx]

**ERN GENTURIS Thematic Group 3: hereditary breast and ovarian cancer syndrome (HBOC) members:**

Sofie Joris, University Hospital Brussels, Brussels, Belgium

Vincent Bours, University Hospital Liege, Liege, Belgium

Lenka Foretova, Masaryk Memorial Cancer Institute, Brno, Czech Republic

Charlotte Lautrup, Aarhus Universitetshospital, Aarhus, Denmark

Thomas Van Overeem Hansen, Rigshospitalet, Copenhagen, Denmark

Tiina Kahre, Tartu University Hospital, Tartu, Estonia

Maria Haanpää, Turku University Hospital, Turku, Finland

Emmanuelle Fourme, Institut Curie, Paris, France

Sophie Frank, Institut Curie, Paris, France

Claude Houdayer, Rouen University Hospital, Rouen, France

Jonas Arnold, Hereditary Cancer Syndrome Center Dresden, Dresden, Germany

Maria C. Roa Bravo, Hereditary Cancer Syndrome Center Dresden, Dresden, Germany

Peter Fasching, Universitätsklinikum Erlangen, Erlangen, Germany

Juliane Hoher, Universitätsklinikum Erlangen, Erlangen, Germany

Anna Lena Burgemeister, Medizinisch Genetisches Zentrum, Munich, Germany

Daniela Turchetti, Azienda Ospedaliero-Universitaria di Bologna, Bologna, Italy

Salvo Testa, Fondaziona Mutagens ETS, Milan, Italy

Maria Antonietta Mencarelli, Azienda Ospedaliero- Universitaria Senese, Siena, Italy

Arvids Irmejs, Pauls Stradins Clinical University Hospital, Riga, Latvia

Vilius Rudaitis, Vilniaus universiteto ligoninės Santaros klinikos, Vilnius, Lithuania

Irma van de Beek, Netherlands Cancer Institute - Antoni van Leeuwenhoek, Amsterdam, the Netherlands

Jan Oosterwijk, University Medical Center Groningen, Groningen, the Netherlands

Christi van Asperen, Leiden University Medical Center, Leiden, the Netherlands

Ingrid Boere, Erasmus Medical Center, Rotterdam, the Netherlands

Margriet Collee, Erasmus Medical Center, Rotterdam, the Netherlands

Linetta Koppert, Erasmus Medical Center, Rotterdam, the Netherlands

Jacek Gronwald, Pomeranian Medical University - University Clinical Hospital no 1, Szczecin, Poland

Isália Miguel, Instituto Português de Oncologia de Lisboa Francisco Gentil, EPE, Lisbon, Portugal

Tamara Hussong Milagre, EVITA Association – Hereditary Cancer (Associação EVITA – Cancro Hereditário), Lisbon, Portugal

Susy Costa, ULS São João, Porto, Portugal

Mateja Krajc, Institute of Oncology Ljubljana, Ljubljana, Slovenia

Darja Molan, Europa Donna Slovenia, Ljubljana, Slovenia

Judith Balmaña, Hospital Germans Trias I Pujol - lnstitut Catala d'Oncologia, Barcelona, Spain

Svetlana Bajalica-Lagercrantz, Karolinska University Hospital, Stockholm, Sweden

Marc Tischkowitz, University of Cambridge, Cambridge, UK
